# The impact of alcohol use disorder and PTSD comorbidity on risk of suicide attempt

**DOI:** 10.1007/s00127-026-03054-y

**Published:** 2026-02-06

**Authors:** Alexis C. Edwards, Sara Larsson Lönn, Ananda B. Amstadter, Mallory Stephenson, Séverine Lannoy, Casey Crump, Jan Sundquist, Kenneth S. Kendler, Kristina Sundquist

**Affiliations:** 1 Virginia Institute for Psychiatric and Behavioral GeneticsDepartment of Psychiatry, Virginia Commonwealth University, Richmond, Box 980126, VA USA; 2 Center for Primary Health Care Research, Lund University, Malmö, Sweden; 3 Department of Human and Molecular Genetics, Virginia Commonwealth University, Richmond, VA USA; 4 Department of Psychology, Virginia Commonwealth University, Richmond, VA USA; 5 Department of Family and Community Medicine, McGovern Medical School, The University of Texas Health Science Center, Houston, USA; 6 Department of Epidemiology, School of Public Health, The University of Texas Health Science Center, Houston, USA; 7 University Clinic Primary Care, Skåne, Sweden

**Keywords:** Alcohol use disorder, Posttraumatic stress disorder, Suicide attempt, Comorbidity

## Abstract

**Background:**

Alcohol use disorder (AUD) and posttraumatic stress disorder (PTSD) are frequently comorbid and are individually associated with increased risk of suicidal behavior. However, whether their comorbidity exacerbates this risk has not been adequately investigated.

**Methods:**

Using a Swedish birth cohort (born 1970–1990; *N* = 799,203–858,983), we employed Aalen’s linear hazards models to evaluate the risk of non-fatal suicide attempt (SA) as a function of registration for AUD and/or PTSD. Models were stratified by sex and considered the temporal ordering of AUD and PTSD. We adjusted for registrations of major depression (MD) and other key covariates.

**Results:**

The overall incidence of SA ranged from 16.52 to 19.29 per 10,000 person-years (PY). In models adjusted for MD and other covariates, PTSD accounted for an additional 12.19–22.09 SA cases per 10,000 PY; the corresponding range for AUD was 43.24–80.04, and the difference in effect size across predictors was more pronounced where AUD preceded PTSD. Comorbidity exacerbated risk: The interaction between PTSD and AUD accounted for 71.13-179.41 additional SA cases per 10,000 PY. In secondary models, interactions between AUD and MD conferred additional SA risk (40.50-127.88 additional SA cases per 10,000 PY), while interactions between PTSD and MD were very weak and, in most cases, negative (-13.26-3.29).

**Conclusions:**

PTSD and AUD are independently associated with SA, but risk is substantially exacerbated among comorbid individuals. While the total effect of these conditions on SA risk is overall comparable across sexes, females whose PTSD precedes AUD are particularly burdened by comorbidity.

**Supplementary Information:**

The online version contains supplementary material available at https://doi.org/10.1007/s00127-026-03054-y.

## Introduction

Suicidal behavior is a leading cause of death globally, accounting for over 49,000 deaths in the US in 2023 [1] and over 720,000 worldwide [2]. For every suicide death in the US in 2022, there were 48 non-fatal suicide attempts (SA) [3]. Together, suicide and non-fatal self-harm cost an average of $510 billion annually in the US from 2015 to 2020 due to lost life years, reduced quality of life, medical spending, and other factors [4]. Although most individuals who survive an attempt do not go on to die by suicide [5], SA remains one of the most consistently implicated correlates of subsequent suicide death [6, 7]. Identifying groups at high risk for SA is therefore a high priority in the effort to reduce the enormous personal, social, and economic costs associated with both fatal and non-fatal suicidal behavior.

Psychiatric illness is common among individuals who engage in suicidal behavior [8, 9]. Here, we focus on the roles of posttraumatic stress disorder (PTSD) and alcohol use disorder (AUD). These conditions are frequently comorbid [10, 11], which is particularly problematic with respect to prognosis and other mental health problems [12]. Both conditions have been repeatedly independently associated with suicidal behavior [12–18]. However, given the high comorbidity and associated difficult clinical course of the disorders, there is a need to jointly investigate their effects in relation to SA.

Importantly, PTSD and AUD are readily integrated into well-established theoretical models of suicidal thoughts and behaviors. PTSD has the potential to exacerbate SA risk by increasing “acquired capability”, a central feature of the Interpersonal Theory and the Three Step Theory [19–21] wherein the threshold for self-harm is reduced by prior exposure to painful or provocative events (PPE). AUD can lead to increased risk of SA through acute intoxication [15, 22, 23], interpersonal conflict [24], increased risk of PPE exposure [24], or other correlates. Both could reflect “dispositional capability” [21] in that they may index genetic liability to SA: Previous studies have reported modest to moderate genetic correlations between PTSD or AUD and suicidal behavior [25, 26].

Given the clinical implications, it is essential to use well-powered samples and varied methods to improve our understanding of how AUD and PTSD contribute to risk for SA. The overarching goal of the current study is to leverage nationwide Swedish registry data to evaluate how comorbidity between AUD and PTSD is related to risk of SA. This data allows us to jointly consider relationships among PTSD, AUD, and SA in a representative birth cohort rather than among selected samples, which have been utilized in much prior research [12, 27–31]. We identified a series of research questions. First, are PTSD and AUD jointly associated with SA risk and is there mediation across disorders? Second, is the temporal ordering of the conditions differentially related to risk of SA? Third, is there a synergistic/non-additive effect of comorbidity; i.e., do we observe more SA cases among comorbid individuals that we would expect based on the main effects of PTSD and AUD? And fourth, do observed associations persist after accounting for another commonly comorbid condition – major depression (MD) – and genetic liability to SA? We address these questions in analyses stratified by sex given evidence of potential sex differences in the relationship between AUD and PTSD [32]. Pursuing these questions could provide additional information to health care providers beyond more universal screening tools.

## Methods

### Study sample

We included everyone born in Sweden between 1970 and 1990. Because we included as a covariate a family genetic risk score for suicide attempt (see below), and such scores are not informative in the absence of family data, only individuals whose parents were also born in Sweden were included.

A series of Swedish nationwide registers were linked using the unique 10-digit identification number assigned at birth to all Swedish residents. The identification number was replaced by a serial number to ensure people’s integrity. The following registers were included: the Multi-Generation Register, the Longitudinal Integrated Database for Health Insurance and Labour Market Studies (LISA), the Hospital Discharge Register, the Prescribed Drug Register, the Outpatient Care Register, the Crime Register, the Swedish Suspicion Register, the Mortality Register. We also used non-registry data from primary health care clinics. Details on the content and available dates are provided in the Supplementary Material. The Regional Ethical Review Board in Lund approved the use of this data.

### Measures

Our outcome variable, non-fatal suicide attempt, was defined from medical records by the following ICD-10 codes: E95, E98, X6, X7, X8, Y1, Y2, Y30, Y31, Y32, Y33, and Y34.

Exposure to PTSD was defined from medical registers using the following codes ICD-9: 308, and 309, and ICD-10: F43.1. F43.0. F43.8, F43.9, F43.2, and F62.0.

Exposure to AUD was assessed using both Swedish medical and criminal registers. From medical registers, we also included medical complications from alcohol use or medication for AUD, and used the following ICD codes: ICD8: 291, 303, 571.0; ICD9: 305 A, 291, 303, 357 F, 425 F, 535D, 571 A, 571B, 571 C, 571D; and ICD 10: ICD-10 codes: F10.1, F10.2, F10.3, F10.4, F10.5, F10.6, F10.7, F10.8, F10.9, E24.4, G31.2, G62.1, G72.1, I42.6, K29.2, K700, K701, K702, K703, K704, K709, K85.2, K86.0, and O35.4. Additionally, AUD was identified using the Prescribed Drug Register by retrieving “Drugs used in alcohol dependence” (Anatomical Therapeutic Chemical [ATC] Classification System code N07BB); this includes disulfiram (N07BB01), acamprosate (N07BB03), naltrexone (N07BB04) or nalmefene (N07BB05). Finally, we identified AUD cases as those convicted for or suspected of at least two alcohol-related crimes according to law 1951:649, paragraph 4 and 4 A and law 1994:1009, Chap. 20, paragraph 4 and 5 from the Swedish Crime Register, and code 3005 and 3201 in the Suspicion register. Most of these crime types include driving or operating a boat while intoxicated, and 99.7% of criminal AUD registrations were related to these codes.

Major depression (MD) was identified by medical register using the following codes: ICD-9: 296B 298 A, 300E, and ICD-10: F32, and F33.

Aggregate genetic liability to SA was assessed using a score, referred to as family genetic risk score for SA (FGRS_SA_), which has been described in detail elsewhere and utilized for a wide range of phenotypes [33, 34]. Comparison to polygenic scores derived using molecular data indicates that FGRS and polygenic scores reflect complementary components of genetic liability [35]. Briefly, we assess genetic risk by an examination of first through fifth degree relatives, correcting for cohabitation effects which could make relatives more similar in their risk for a given outcome (here, SA). The FGRS is a relative measure which is operationalized as a continuous, standardized indicator of aggregate genetic risk, so that one unit corresponds to one standard deviation in the population.

Individual socioeconomic status was assessed with the parents’ highest level of education and categorized into low (compulsory school only), mid (upper secondary school), high (university level), and missing. Parental education was used to ensure that the individual’s socioeconomic status was assessed prior to the exposure and was consistent across time.

### Statistical models

The exposure variables were included as time dependent in all analyses and consequently the follow-up times differed between exposure groups. We therefore present total follow-up time and incidence rates for each predictive variable.

We utilized additive Aalen models for all analysis to assess the effects of exposures and interactions on consecutive SA on an additive scale, which is preferred over a multiplicative model for outcomes of public health relevance [36]. We censored at death, emigration or end of follow-up (December 31, 2018) whichever came first.

Two separate datasets were created to enable us to estimate associations with SA depending on the temporal ordering of PTSD and AUD. In one dataset, we censored at the time of PTSD registration if preceded by an AUD registration, and in the other we censored at the time of AUD registration if preceded by a PTSD registration. Analyses for these datasets were executed in parallel. Models that contain only the primary predictors of interest (PTSD and AUD) or their interaction have the prefix “1” (i.e., 1a, 1b, and 1c). Those models are then expanded to include sociodemographic covariates, which are denoted with the prefix “2” (2a, 2b, 2c). Model 3a further includes a key psychiatric covariate (MD), while model 3b also includes FGRS_SA_. Finally, Models 4a-4e explore expansions of Model 3a to consider secondary hypotheses regarding interactions among psychiatric exposures.

To obtain an approximate estimate of the proportion of the effect of PTSD that was mediated through AUD we calculate the ratio between the reduction of the association between PTSD and SA when AUD is added to the model, and the total effect. The corresponding models are run for analysis of AUD preceding a possible PTSD diagnosis. All analyses were stratified by sex and we present the result as number of cases per 10,000 PY. We inferred a statistically significant effect if 95% confidence intervals (CIs) for a variable of interest, with respect to the number of additional cases accounted for, did not include 0.

All analyses were conducted in RStudio version 2024.12.0 [37]. The Aalen models were run using the *aalen* function in the *timereg* package [38].

## Results

### Descriptive statistics

Table 1 provides descriptive statistics for the sample, stratified by sex, and separately for analyses where AUD did not precede a PTSD registration versus where PTSD did not precede an AUD registration. The analytic sample size range was *N* = 799,203–838,983. The mean (SD) birth year for males was 1979.97 (6.26) and for females was 1979.95 (6.27). Across sexes and datasets, the overall incidence of SA ranged from 16.52 to 19.29 per 10,000 PY.

**Table 1:** Descriptive statistics for the analytic sample. The top panel represents the “posttraumatic stress disorder (PTSD) first” analysis which required that any PTSD registration occurred before a registration for alcohol use disorder (AUD) diagnosis; if an AUD registration occurred first, the subjects were censored at the date of the PTSD registration. The inverse approach was applied for the bottom panel, which represents the sample for the “AUD first” analysis

	Males			Females		
	Number (%)	Follow-up time (person years)	Incidence rate (per 10,000 person years)	Number (%)	Follow-up time (person years)	Incidence rate (per 10,000 person years)
***PTSD first***						
Total N (row %)	858,983 (51.8%)	15,412,959.9	18.75	799,269 (48.20%)	14,342,418.1	16.54
Low parental education	68,777 (8.01%)	1,457,035.5	18.74	64,352 (8.05%)	1,369,213.2	15.73
Mid parental education	394,257 (45.9%)	7,131,084.5	20.10	368,322 (46.08%)	6,671,060.2	17.02
High parental education	395,949 (46.1%)	6,824,839.9	17.35	366,595 (45.87%)	6,302,144.7	16.21
PTSD registrations	83,013 (9.66%)	477,870.89	61.10	195,642 (24.48%)	1,128,466.57	46.59
AUD registrations	38,512 (4.48%)	372,538.42	95.29	14,616 (1.83%)	124,459.69	108.79
MD registrations	106,231 (12.37%)	703,700.58	76.88	179,387 (22.44%)	1,268,752.92	63.31
With AUD and PTSD registrations	3,643 (0.42%)	19,085.75	247.30	3,075 (0.38%)	14,528.00	300.80
With PTSD and MD registrations	33,344 (3.88%)	161,483.32	93.82	92,128 (11.53%)	458,940.92	33.01
With AUD and MD registrations	14,652 (1.71%)	81,605.76	168.86	8,475 (1.06%)	46,598.22	295.72
With PTSD, AUD, and MD registrations	2,368 (0.28%)	10,702.89	282.17	2,354 (0.29%)	10,073.67	299.79
***AUD first***						
Total N (row %)	858,931 (51.74%)	15,397,298.78	19.29	799,203 (48.26%)	143,32,987.53	16.52
Low parental education	68,769 (8.01%)	1,455,223.25	19.41	64,347 (8.05%)	1,368,427.65	15.80
Mid parental education	394,228 (45.9%)	7,123,053.82	20.72	368,289 (46.08%)	6,665,996.38	17.00
High parental education	395,934 (46.1%)	6,819,021.7	17.77	366,567 (45.87%)	6,298,563.49	16.16
AUD registrations	34,869 (4.06%)	353,452.67	129.69	11,541 (1.44%)	109,931.69	163.19
PTSD registrations	88,892 (10.35%)	488,824.89	55.91	199,539 (24.97%)	1,134,579.95	40.68
MD registrations	105,846 (12.32%)	694,528.25	81.35	179,180 (22.42%)	1,261,421.46	62.65
With AUD and PTSD registrations	5,588 (0.65%)	27,785.53	252.29	3,511 (0.44%)	17,395.34	208.68
With AUD and MD registrations	12,284 (1.43%)	70,902.88	253.45	6,121 (0.77%)	36,524.55	254.9
With PTSD and MD registrations	36,271 (4.22%)	165,623.67	85.19	94,475 (11.82%)	461,254.61	58.6
With AUD, PTSD, and MD registrations	3,237 (0.38%)	13,661.83	303.77	2,405 (0.30%)	10,374.85	245.79

Within the dataset constructed for assessing the effect of PTSD which was not preceded by AUD^1^, the mean age of onset for PTSD was 33.09 (7.02) for males and 33.24 (6.93) for females. For AUD, the mean age of onset was 28.44 (7.78) for males and 28.79 (8.59) for females. The mean time elapsed from PTSD to AUD registration was 3.82 (3.97) years in males and 4.46 (4.35) years in females.

Within the dataset constructed for examining the effect of AUD which was not preceded by PTSD, the mean age at first AUD registration was slightly lower than in the other dataset: 27.86 (7.68) for males and 27.16 (8.38) for females. Mean age at PTSD registration was 33.17 (6.98) for males and 33.25 (6.92) for females. Progression from AUD to PTSD registration was longer than for PTSD to AUD: 7.09 (6.35) years for males and 7.21 (6.68) for females.

### Models with PTSD preceding AUD

**Fig. 1:**
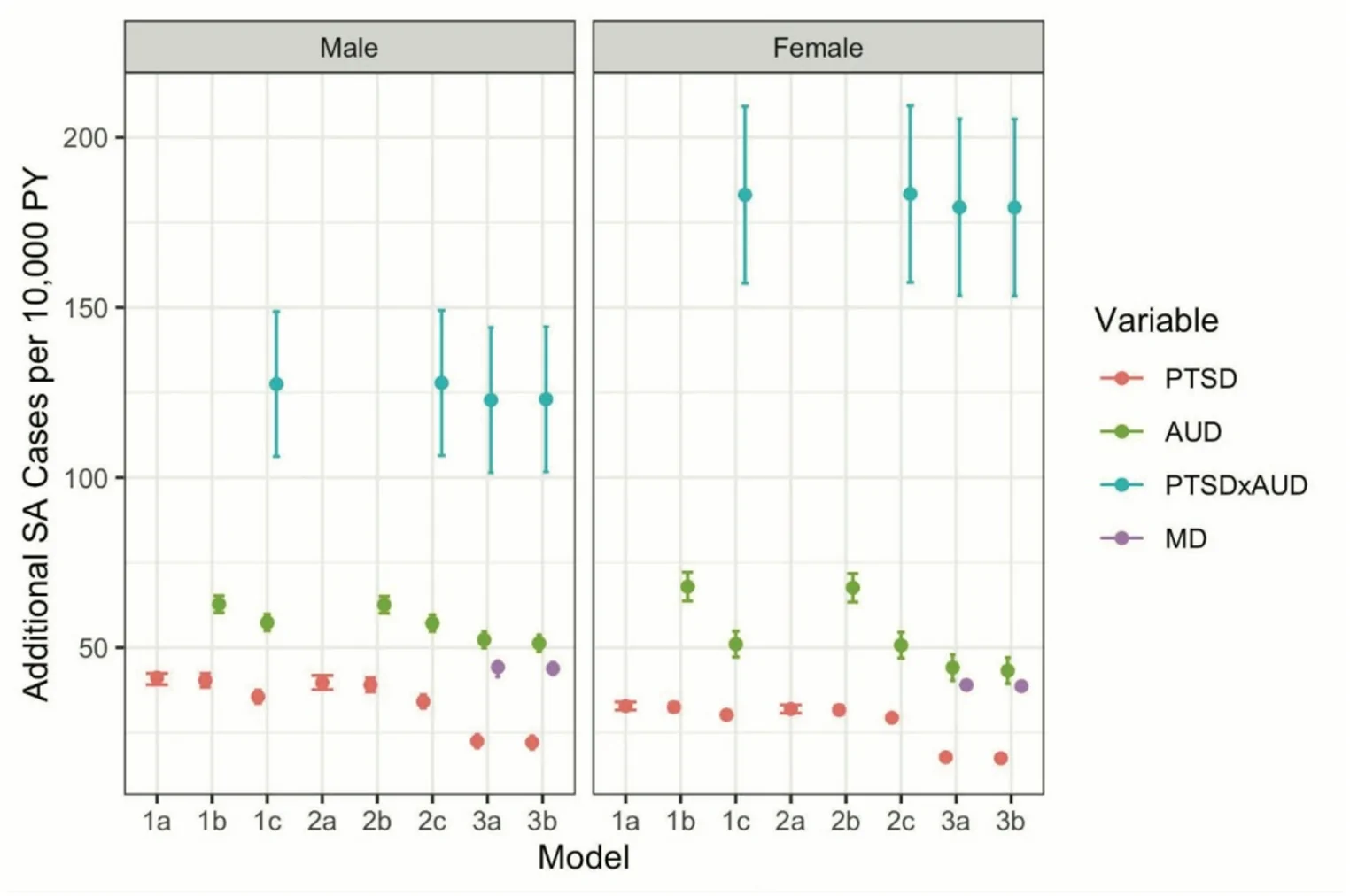
Results from models estimating the effects of post-traumatic stress disorder (PTSD) and alcohol use disorder (AUD) on risk of suicide attempt, where registrations for PTSD must have preceded registrations for AUD. Results are presented as the number of additional suicide attempt cases per 10,000 person years, with 95% confidence intervals

*Model 1.* We first tested crude associations between PTSD registrations and risk of SA. In models with no additional covariates, a PTSD registration was associated with 41.14 additional new SA cases in males and 32.83 in females (Supplementary Tables 1 and Fig. 1). The addition of AUD (Model 1b) and an interaction term between PTSD and AUD (Model 1c) resulted in only minor shifts in the number of additional SA cases attributable to PTSD. For both sexes, the effect of AUD was stronger than that of PTSD. The synergistic effect (operationalized in Model 1c as the interaction term) was higher than the PTSD and AUD main effects, and was higher for females than males: 127.50 additional SA cases in males and 183.14 in females.

*Model 2.* We next included sociodemographic covariates in Models 2a-2c. Estimates for the primary predictors (PTSD, AUD, PTSD⋅AUD) were similar to those in Models 1a-1c (Table 2; Fig. 1). Complete results including estimates for sociodemographic covariates are provided in Supplementary Table 1.

**Table 2:** Results from models estimating the effects of posttraumatic stress disorder (PTSD) and alcohol use disorder (AUD) on risk of suicide attempt, where registrations for PTSD must have preceded registrations for AUD. Results are presented as the number of additional suicide attempt cases per 10,000 person years, with 95% confidence intervals

Males	Model 2a	Model 2b	Model 2c	Model 3a	Model 3b
PTSD	39.75 (37.68, 41.81)	39.03 (36.97, 41.09)	34.16 (32.20, 36.12)	22.46 (20.49, 24.42)	22.09 (20.13, 24.06)
AUD		62.58 (60.10, 65.07)	57.18 (54.76, 59.61)	52.31 (49.91, 54.71)	51.26 (48.87, 53.66)
PTSD⋅AUD			127.82 (106.48, 149.16)	122.82 (101.48, 144.16)	123.04 (101.70, 144.38)
MD				44.23 (41.42, 46.05)	43.84 (42.02, 45.66)
FGRS_SA_					2.69 (2.55, 2.83)
**Females**					
PTSD	31.95 (30.73, 33.16)	31.67 (30.46, 32.88)	29.36 (28.18, 30.54)	17.77 (16.58, 18.96)	17.45 (16.26, 18.64)
AUD		67.61 (63.41, 71.80)	50.68 (46.88, 54.49)	44.12 (40.32, 47.91)	43.23 (39.44, 47.03)
PTSD⋅AUD			183.40 (157.40, 209.39)	179.48 (153.48, 205.48)	179.41 (153.41, 205.41)
MD				38.99 (37.76, 40.23)	38.64 (37.41, 39.88)
FGRS_SA_					2.57 (2.38, 2.65)

PTSD=post-traumatic stress disorder; AUD=alcohol use disorder; MD=major depression; FGRS_SA_=family genetic risk score for suicide attempt

*Model 3.* In Model 3a we added MD as a covariate, accompanied by FGRS_SA_ in Model 3b. In both sexes, MD accounted for a similar number of excess SA cases as AUD. FGRS_SA_ had a small but significant effect. As observed in Models 1c and 2c, the PTSD⋅AUD interaction in Model 3b had a strong effect, accounting for 123.04 additional SA cases in males and 179.41 additional cases in females.

Across Models 1–3, comparing submodels “a” and “b”, there was little evidence that the effect of primary PTSD on SA risk was mediated by AUD: In each case, inclusion of AUD in those models decreased the main effect of PTSD by < 2%.

### Models with AUD preceding PTSD

**Fig. 2:**
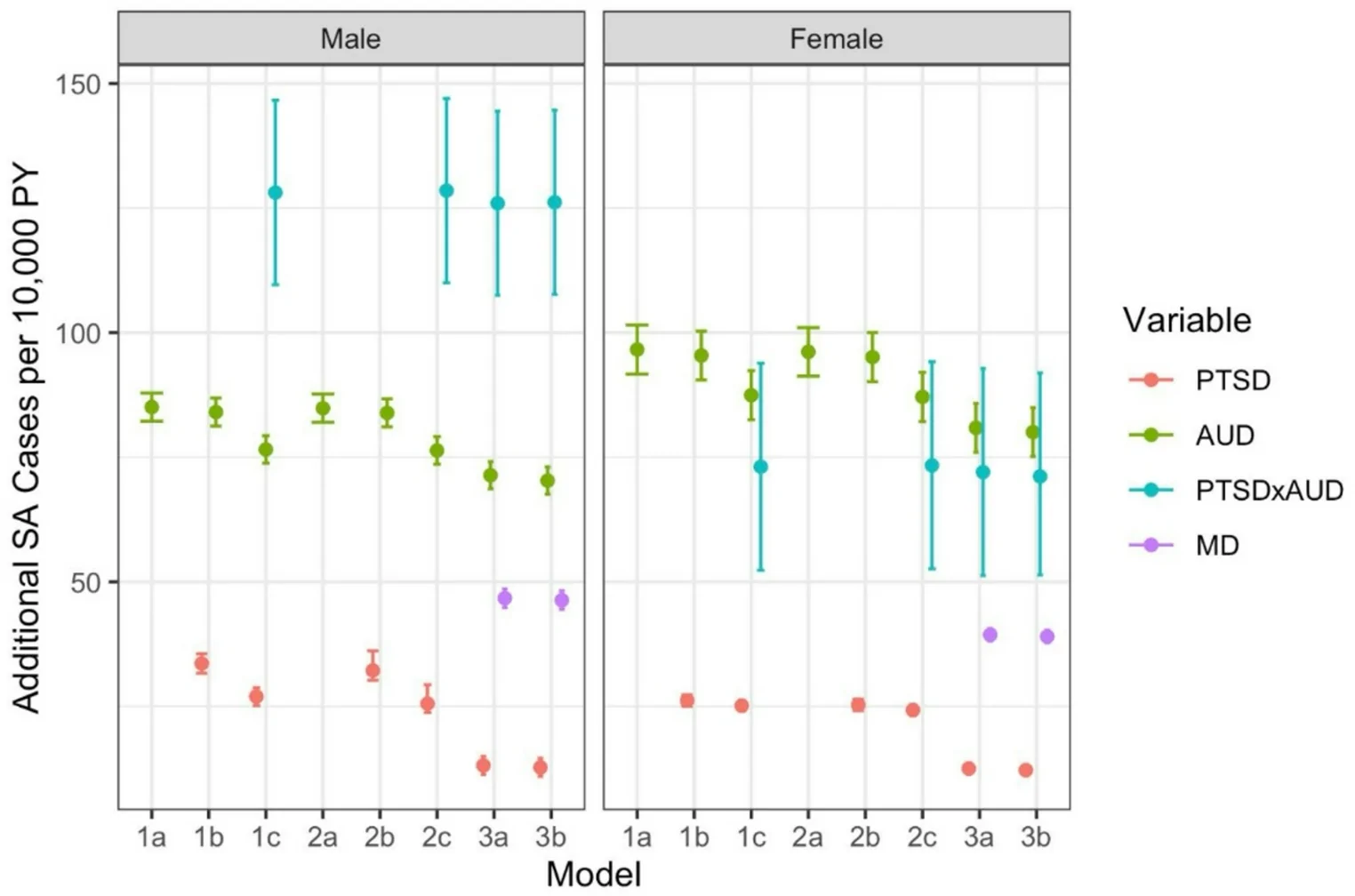
Results from models estimating the effects of post-traumatic stress disorder (PTSD) and alcohol use disorder (AUD) on risk of suicide attempt, where registrations for AUD must have preceded registrations for PTSD. Results are presented as the number of additional suicide attempt cases per 10,000 person years, with 95% confidence intervals

*Model 1.* Associations between AUD and SA risk were considerably stronger in models where AUD was not preceded by PTSD (Supplementary Tables 2 and Fig. 2). In Model 1a, an AUD registration was associated with 85.06 additional SA cases per 10,000 PY in males and 96.61 in females. Accounting for subsequent PTSD registrations and the interaction between AUD and PTSD attenuated these estimates slightly, to 76.55 in males and 87.45 in females. Consistent with models where PTSD preceded AUD, the effect of PTSD was lower than that of AUD. The PTSD⋅AUD interaction was associated with 128.10 additional SA cases in males and 73.08 in females.

*Model 2.* Adjusting for sociodemographic covariates had little impact on the main effects of AUD or PTSD, or on their interaction (Table 3; Fig. 2).

**Table 3:** Results from models estimating the effects of posttraumatic stress disorder (PTSD) and alcohol use disorder (AUD) on risk of suicide attempt, where registrations for AUD must have preceded registrations for PTSD. Results are presented as the number of additional suicide attempt cases per 10,000 person years, with 95% confidence intervals

Males	Model 2a	Model 2b	Model 2c	Model 3a	Model 3b
AUD	84.83 (82.01, 87.66)	83.89 (81.08, 86.70)	76.35 (73.59, 79.11)	71.38 (68.64, 74.11)	70.31 (67.58, 73.04)
PTSD		32.20 (30.26, 36.14)	25.55 (23.77, 29.33)	13.14 (11.34, 14.95)	12.75 (10.95, 14.56)
PTSD⋅AUD			128.50 (110.00, 147.00)	125.96 (107.49, 144.43)	126.16 (107.69, 144.64)
MD				47.45 (45.56, 49.34)	46.31 (44.45, 48.17)
FGRS_SA_					2.81 (2.67, 2.95)
**Females**					
AUD	96.15 (91.24, 101)	95.09 (90.18, 100.00)	87.10 (82.17, 92.03)	80.89 (75.98, 85.81)	80.04 (75.13, 84.95)
PTSD		25.29 (24.16, 26.42)	24.24 (23.14, 25.34)	12.51 (11.39, 13.64)	12.19 (11.06, 13.31)
PTSD⋅AUD			73.35 (52.57, 94.13)	72.02 (51.26, 92.79)	71.13 (51.36, 92.90)
MD				39.37 (38.13, 40.61)	39.02 (37.78, 40.26)
FGRS_SA_					2.51 (2.37, 2.64)

PTSD=post-traumatic stress disorder; AUD=alcohol use disorder; MD=major depression; FGRS_SA_=family genetic risk score for suicide attempt

*Model 3.* Including MD as a covariate attenuated the effects of both AUD and PTSD, (Table 3; Fig. 2). For both sexes, the number of additional SA cases accounted for by PTSD was reduced by approximately half from Model 2c to Model 3b. The shift in AUD’s effect was less pronounced: In males, it was reduced from 76.35 to 71.38, and in females from 87.10 to 80.89. MD itself accounted for 46.31 additional SA cases in males and 39.02 in females (Model 3b). This contrasts with the results above: If an AUD registration preceded a PTSD registration (rather than vice versa), the effect size of MD was considerably lower than that of AUD. Effects of the PTSD⋅AUD interaction were only slightly attenuated for both sexes. Again, the number of additional SA cases attributable to FGRS_SA_ was small but significant. Complete results including estimates for sociodemographic covariates are provided in Supplementary Table 2.

As above, when comparing submodels “a” and “b” in Models 1–3, the main effect of AUD was only slightly attenuated by further accounting for PTSD: Reductions in the number of estimated additional SA cases as a function of AUD status were < 2%.

### Secondary analyses

Given the strong joint effects of PTSD and AUD, we elected to conduct a series of *post hoc* tests. Our aim was to determine whether the strong interaction effect was specific to PTSD and AUD *versus* being generalizable to interactions between two psychiatric conditions. In particular, we were interested in the possibility that the combination of an externalizing disorder (AUD) with an internalizing disorder (PTSD or MD) was more impactful than the combination of two internalizing disorders (PTSD and MD). We therefore evaluated the effects of two alternative interaction terms, namely PTSD⋅MD and AUD⋅MD, within the context of Model 3a (i.e., prior to including FGRS_SA_ as a covariate). As above, models were run separately depending on which primary exposure of interest – PTSD *versus* MD – occurred first; this series was referred to as Model 4a-e.

**Fig. 3:**
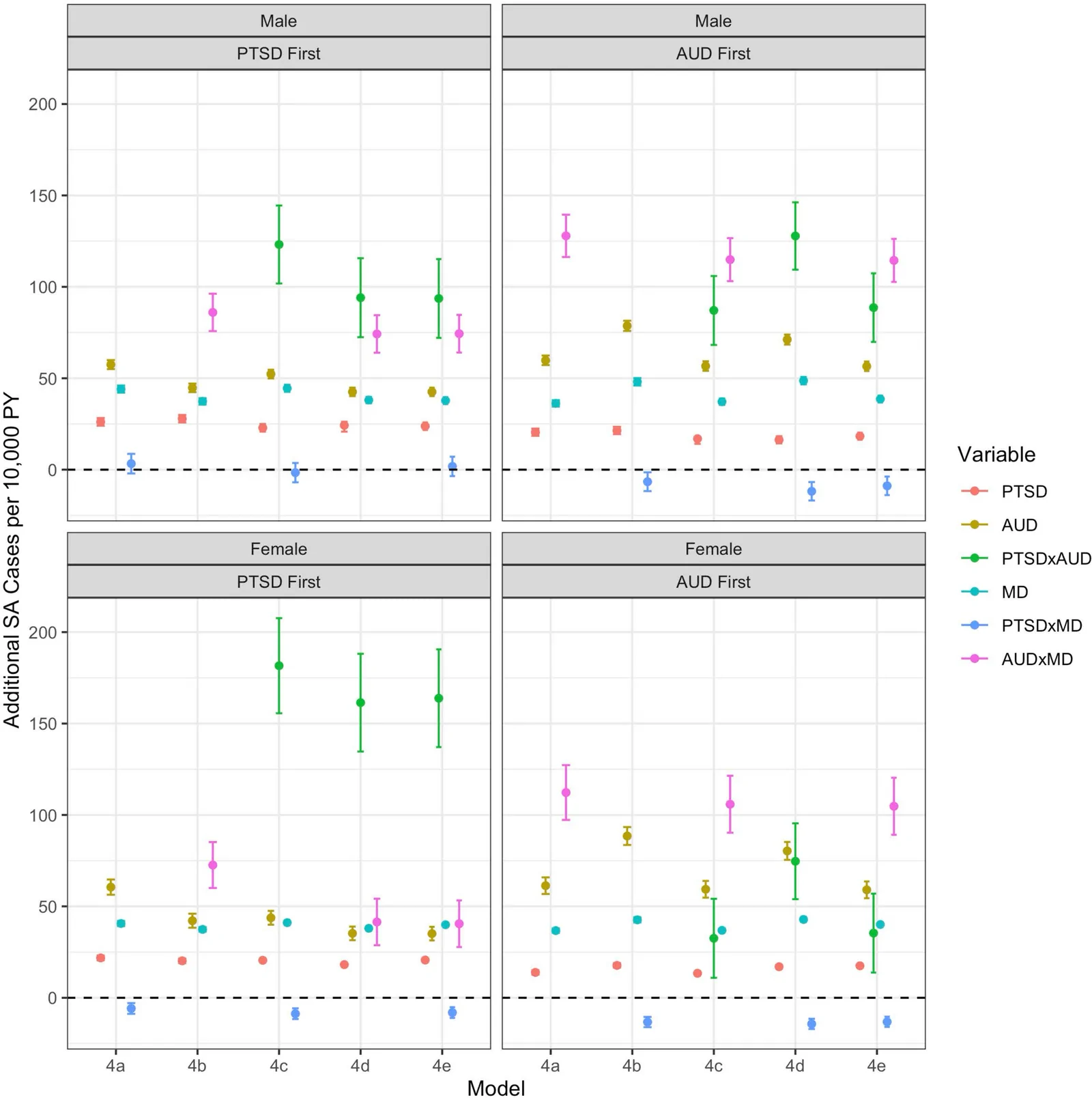
Results from models estimating interactions between post-traumatic stress disorder (PTSD), alcohol use disorder (AUD), and major depression (MD). Results are presented as the number of additional suicide attempt cases per 10,000 person years, with 95% confidence intervals. Separate models were executed for males (top panels) and females. Results in panels on the left are derived from models wherein a PTSD registration could not have been preceded by an AUD registration; panels on the right represent results from models wherein an AUD registration could not have been preceded by a PTSD registration

As shown in Supplementary Tables 3 and Fig. 3, the impact of psychiatric comorbidity varies considerably as a function of the conditions involved, the temporal ordering of PTSD and AUD, and – to a lesser extent – sex. Most notably, the effect of the PTSD⋅MD term was either not significant or was weakly negative. Interactions between AUD and MD were consistently positive, but were of less impact where PTSD (rather than AUD) was the initial registration. Finally, in models that included both PTSD⋅AUD and AUD⋅MD terms (Model 4c if AUD was the initial registration, and Model 4e across both conditions), the number of additional SA cases per 10,000 PY was generally higher for the AUD⋅MD term.

## Discussion

With these analyses, we aimed to investigate the impact of PTSD and AUD, two commonly comorbid conditions, on risk of non-fatal SA. Although prior work has demonstrated independent risk associated with each condition, fewer studies have examined the impact of comorbidity. Important extensions to prior research in this area include our consideration of potential differences in the impact of PTSD or AUD depending on which condition occurred first; our inclusion of MD, which frequently co-occurs with both PTSD and AUD, and of aggregate genetic liability to SA given its modest heritability; and our explicit evaluation of the interaction on the additive scale between PTSD and AUD on SA risk. Key findings include: (i) the impact of AUD on SA risk is generally higher than that of PTSD; (ii) the magnitude of AUD’s impact is considerably higher if it precedes a PTSD registration rather than follows it; (iii) the interaction between PTSD and AUD is consistent in males regardless of which condition occurs first, while in females, it is approximately twice as strong when PTSD is registered first; and (iv) MD has a substantial main effect on SA risk, minimally attenuates the main effect of AUD, and more strongly attenuates the effect of PTSD.

The observation that AUD accounts for more SA cases than PTSD adds to a growing literature exploring the impact of these conditions [39–41]. In a population-based US sample in which the dimensionality of PTSD was considered, the relative effect of AUD versus facets of PTSD on SA risk was inconsistent [42], while two studies of US veterans found that those with PTSD-only were at higher risk of a lifetime SA than those with AUD-only [12, 27]. Discrepancies between current and previous findings could be attributable in part to disparate methodological approaches. Sample differences could also play a role: Much of the extant literature on PTSD and AUD focuses on military or veteran, rather than population-based, samples. The nature of the exposure that precipitated PTSD development likely differs across these groups (i.e., combat versus civilian traumas such as accidents, disasters, and interpersonal violence). Where possible, future study designs should distinguish among types of traumatic exposures to help disentangle these associations and consider whether specific subpopulations are differentially impacted by PTSD-AUD comorbidity.

We pursued analyses that considered the temporal ordering of PTSD and AUD primarily to determine whether we could detect a subgroup with particularly high SA risk. In Model 2b, the total number of additional SA cases per 10,000 PY attributable to PTSD and AUD was slightly higher if AUD preceded PTSD. However, expanding our perspective to consider both main effects and the interaction between AUD and PTSD in Model 2c, a more nuanced picture emerged: The total impact was 219.16 additional SA cases in males and 263.43 in females if PTSD occurred first, while the corresponding estimates if AUD occurred first were 230.45 in males and 184.69 in females. Thus, among males we observed a very minor increase in SA risk for those whose AUD preceded their PTSD, while females whose PTSD precedes AUD were at strikingly higher risk. These figures shifted only slightly when accounting for comorbid MD. While we observed considerable deviation from additivity in nearly every instance, our findings suggest that AUD prevention efforts among females with PTSD could have an especially pronounced effect on reducing SA. Earlier work by our group found that for both sexes the risk of PTSD on future AUD is highest in the first five years after diagnosis, with substantial attenuation of risk thereafter, highlighting the importance of timing in prevention work [43].

We did not conduct formal mediation models; however, mediation can be informally examined by comparing the effect sizes of PTSD or AUD in Model 2a, which includes only one or the other, to those in 2b, where the effect of the condition whose registration occurred first is potentially attenuated by the temporally secondary condition. Across both temporal arrangements (PTSD first versus AUD first) and sexes, the addition of the second condition had only minimal impact on the effect of the first: <2% of the total effect was indirect. This contrasts with a previous report that AUD mediated some aspects of the PTSD ◊ SA association [42]. Thus, despite evidence supporting self-medication of PTSD symptoms through problematic alcohol use [44], the current results do not suggest that that pathway accounts for SA risk among comorbid individuals. Similarly, although those with AUD are at increased risk of exposure to traumatic events and subsequent development of PTSD [45], the effects of AUD and PTSD on SA risk are largely independent of one another.

The primary motivations for including MD in our adjusted models were to account for its well-established association with suicidal behavior [16, 46–48], compare its relative impact to that of AUD and PTSD, and confirm that the associations between AUD or PTSD and SA were robust to its inclusion. The inclusion of MD in the models was associated with a modest attenuation of the effects of AUD and a more moderate attenuation of the effects of PTSD; however, the main effects remained significant in these models. Further, the inclusion of MD did not attenuate the effects of the PTSD⋅AUD interaction across models. In most cases, the impact of AUD was significantly stronger than that of MD, consistent with some [16] but not all [49–51] prior studies of suicidality. This could be attributable in part to our detection of MD across a range of severity, while most AUD cases detected in registry data are severe. In turn, MD’s effect was stronger than that of PTSD. Only among females whose PTSD registration could not have been preceded by an AUD registration were the effects of MD and AUD equivalent.

We next scrutinized the change in AUD and PTSD’s effect sizes from Models 2c to 3a as an informal interpretation of mediation by MD. We observed that the total effect of AUD on SA was reduced by 6–13% when MD was added to the model, while the corresponding attenuation of PTSD’s effect was far more pronounced, ranging from 34 to 49%. Thus, in contrast to the parallel estimates for AUD and PTSD’s informal mediation of one another described above, MD can account for a non-trivial proportion of both AUD and PTSD’s impact. However, main effects of PTSD and AUD remained, underscoring the unique contributions of each condition to SA risk. This implies that treatment of MD, while certainly beneficial, is unlikely to sufficiently reduce SA risk among individuals comorbid for PTSD and/or AUD.

Including MD in the model also allowed us to evaluate whether the interaction between AUD and PTSD reflected risk specific to those conditions, versus whether it reflected how externalizing and internalizing comorbidity exacerbated SA risk. Recent research demonstrates that PTSD is genetically transmitted alongside internalizing more strongly than externalizing disorders [52, 53]. Our question was addressed by comparing three interaction terms – PTSD⋅AUD, AUD⋅MD, and PTSD⋅MD – within the Model 4 series. We observed positive and strong deviations from additivity for internalizing-externalizing interactions, but weakly negative or non-significant negative deviations from additivity for the PTSD⋅MD interaction. Thus, the effect of an internalizing disorder (PTSD or MD) is far less impactful among individuals who already have another internalizing disorder. Both retain a significant main effect, but their joint impact is less than would be predicted based on the sum of the main effects. In contrast, the combination of an externalizing disorder (AUD) and an internalizing disorder (PTSD and/or MD) consistently exacerbates SA risk. One implication of this finding is that the nature of comorbidity is important in SA risk estimation: Clinicians should consider patients/clients with comorbid internalizing and externalizing conditions to be a particularly high-risk subgroup warranting careful screening for suicidality.

Our findings highlight deficits in the current literature that should be addressed in future research. First, it will be important to pursue explanations underlying the sex differences identified here and in prior research on AUD and PTSD [43], including the role of exposure to specific trauma types, which may differ by sex in both in prevalence and impact [54]. Second, the impact on suicidality risk of strategies that target individuals comorbid for AUD and PTSD is understudied. Evidence suggests that integrated treatments for PTSD and AUD are safe (i.e., do not increase risk for STB) and effective. COPE (Concurrent Treatment of Substance Use Disorders and PTSD using Prolonged Exposure), the only integrated treatment that employs trauma-focused techniques, has been shown to reduce both PTSD and AUD severity [55]. COPE trials have included individuals with suicidal ideation and prior attempts (e.g., 52.4% of participants reported a lifetime suicide attempt, and 9.7% reported a past year attempt [56]). Although COPE has been found to decrease other psychopathology targets beyond PTSD and AUD, including depressive symptoms, to date, no trials have specifically examined effects on suicidal thoughts and behaviors.

Finally, an in-depth investigation of the mechanisms by which PTSD and AUD jointly increase SA risk should be the focus of future studies. Prior work suggests that behavioral and neurocognitive features of individuals with both conditions – specifically, impulsivity and cognitive inflexibility – may contribute to increased risk of suicidality [28]. Furthermore, AUD and PTSD fit well within contemporary models of suicide risk, such as the Interpersonal Theory of Suicide [20] and the Three-Step Theory [21]: Both could contribute to a sense of interpersonal disconnection; AUD could be an indicator of dispositional capability (e.g., an underlying propensity for impulsive behaviors); and some traumatic exposures leading to PTSD could lead to acquired capability.

Although an epidemiologic perspective is the focus of this report, it is important to consider a clinical perspective as well. Recent guidance [57] issued within the United Kingdom recommends against risk stratification by health care providers interfacing with individuals who have self-harmed, arguing for “psychosocial assessment” rather than “risk assessment” [58]. Indeed, suicidal behavior is notoriously difficult to predict with high confidence: Although recent approaches applying machine learning suggest some improvements [59], more traditional methods typically perform poorly [60, 61]. Thus, it would be inappropriate to use the current findings in isolation to characterize individuals (with or without a history of self-harm) as warranting or not warranting clinical care or other intervention. As suggested by the aforementioned guidance, an individual’s particular circumstances must be considered in such determinations. Consistent with the NICE guidelines, the current findings suggest that PTSD and AUD could be considered for assessment by clinicians during a thorough psychosocial assessment.

Our findings should be considered through the lens of some limitations. First, while our analyses were conducted in a large Swedish birth cohort, results might not replicate in different sociocultural contexts. Sweden has a robust social safety net system, including universal health care, which could facilitate treatment-seeking for PTSD and/or AUD relative to in other countries. Second, we relied on national registry resources which, while free from recall bias and other limitations of self-reported data, are likely to largely reflect more severe cases of psychiatric conditions; e.g., suicide attempts that did not require medical attention may not be captured. Third, AUD is a chronic, recurring condition that may be diagnosed only after an individual has suffered from its symptoms for some time. This could have impacted estimates from our PTSD-first analyses, as it is possible that individuals developed AUD prior to PTSD but were not registered for it until later. To bolster confidence in the current findings, our research questions should be addressed in independent samples, preferably through the use of additional assessment approaches, such as self-reports, and by applying complementary methods, including formal mediation models. Additionally, our reliance on registration codes for PTSD-only preclude our ability to separate out the direct impact of traumatic events from that of PTSD on SA risk, a critical direction for future work. Finally, although our findings indicate that AUD and PTSD are robustly associated with SA, these factors do not operate in isolation; health care providers concerned about SA risk should consider the presence of these conditions within the larger context of patients’ psychosocial circumstances.

In summary, we report robust evidence in a statistically powerful, population-based sample that AUD and PTSD independently impact risk of suicide attempt, and that their joint effect (seen in a deviation from additivity) is considerable. These effects were attenuated by accounting for MD but remained significant. Our findings underscore the importance of considering comorbidity of multiple psychiatric disorders, and in particular suggest that risk is amplified among individuals suffering from both internalizing and externalizing disorders. Furthermore, the magnitude of SA risk varies as a function of sex and the temporal order of conditions. While the utility of universal suicide risk screening and risk stratification is the topic of ongoing discussion [57, 62, 63], our findings may be used to inform a more nuanced understanding of suicidality risk in clinical contexts.

## Supplementary Information

Below is the link to the electronic supplementary material.


Supplementary Material 1



Supplementary Material 2



Supplementary Material 3



Supplementary Material 4


## Data Availability

The datasets analyzed during the current study are not publicly available due to restrictions by Statistics Sweden.

## Footnotes

1 Individuals whose AUD registration preceded a PTSD registration were included in this dataset but were censored at the time of PTSD registration. Hence, the mean age of AUD onset is younger than the mean PTSD onset.
