# Supplementary material for: The impact of alcohol use disorder and PTSD comorbidity on risk of suicide attempt
Source: Soc Psychiatry Psychiatr Epidemiol. 2026 Feb 6;61(8):1647–59. doi: 10.1007/s00127-026-03054-y (PMC13340058; doi:10.1007/s00127-026-03054-y)
Supplement: Supplementary file 1 [file 127_2026_3054_MOESM1_ESM.pdf]

The impact of alcohol use disorder and PTSD comorbidity on risk of suicide attempt

Supplementary Material

Supplementary Methods

Details of the Swedish nationwide registers utilized are as follows:

- Multi-Generation Register, containing information about sex, year of birth, and parents for individuals born after 1932 or if immigrated together with their parents
- Longitudinal Integrated Database for Health Insurance and Labour Market Studies (LISA), containing information about education and social welfare recipients from 1990 to 2018
- Hospital Discharge Register, containing hospitalizations for Swedish inhabitants from 1964 to 2018
- Prescribed Drug Register, containing all prescriptions in Sweden picked up by patients from July 2005 to 2018
- Outpatient Care Register, containing information from all outpatient clinics from 2001 to 2018
- Crime Register, containing national complete data on all convictions in lower court from 1973 to 2018
- Swedish Suspicion Register, containing national data on individuals strongly suspected of crime from 1998 to 2018
- Mortality Register with dates and causes of death from 1952 until 2018.
- Medical diagnoses from regional Primary Health Care clinics in Region Stockholm (2003 - 2018), Uppsala (2005 - 2018), Södermanland (1997 - 2018), Östergötland (1990 - 2018), Jönköping (2008 - 2018), Kronoberg (2006 - 2018), Kalmar (2007 - 2018), Gotland (2011 – 2018), Blekinge (2009 - 2018), Skåne (1989 - 2018), Halland (2007 - 2018), Värmland (2005 - 2018), Örebro (2013 to 2006), Västmanland (2014 to 2018), Dalarna (2005 - 2018), Gävleborg (2010 - 2018), Västernorrland (2010 - 2018) , Västerbotten (1992- 2018), Norrbotten (2001 - 2018) and Västra Götalandsregionen (2000 - 2018). The time periods varied due to the regions’ different timing of digitalizing of the patient records.
